# Different glaucoma progression rates by age groups in young myopic glaucoma patients

**DOI:** 10.1038/s41598-024-53133-w

**Published:** 2024-01-31

**Authors:** Eun Jung Lee, Dongyoung Lee, Min-Ji Kim, Kyunga Kim, Jong Chul Han, Changwon Kee

**Affiliations:** 1 Department of Ophthalmology, Samsung Medical Center, Sungkyunkwan University School of Medicine, Seoul, South Korea; 2 Biomedical Statistics Center, Research Institute for Future Medicine, Samsung Medical Center, Seoul, South Korea; 3 Department of Ophthalmology, Kim’s Eye Hospital, Konyang University College of Medicine, Seoul, South Korea

**Keywords:** Optic nerve diseases, Eye diseases

## Abstract

We aimed to investigate the age-related glaucoma progression rates in myopic normal tension glaucoma (NTG). In this long-term retrospective cohort (7.2 ± 3.5 years), we grouped patients based on their age at initial presentation: group A (age < 30 years, 60 eyes), group B (30 ≤ age < 40, 66 eyes), and group C (40 ≤ age < 50 years, 63 eyes). We used a linear mixed-effects model to estimate retinal nerve fiber layer (RNFL) defect width enlargement rates. Group A showed a significantly faster rate of RNFL defect progression (3.01 ± 1.74°/year) than those of groups B and C (2.05 ± 1.55°/year and 2.06 ± 1.29°/year, *P* = 0.004 and 0.002). The difference was more marked when calculated for the first 10 years of follow-up in group A, B, and C (3.95 ± 2.70°/year, 2.39 ± 1.64°/year, and 1.98 ± 1.31°/year), and between the periods of age < 30 years, 30 ≤ age < 40 years, and 40 ≤ age < 50 years within group A. This is the first evidence of rapid glaucoma progression in the young adulthood and stabilization in older age in myopic NTG. Clinicians should consider the potentially aggressive course of glaucoma, especially in younger patients with myopic NTG, in contrast to the general slow progression in adulthood.

## Introduction

Glaucoma is a multifactorial optic neuropathy characterized by progressive retinal ganglion cell loss and visual field (VF) damage^1^. The rising prevalence of myopia globally^2^ has made myopic glaucoma, particularly normal tension glaucoma (NTG), a growing concern. Moreover, myopic NTG tends to develop at younger ages compared to the general glaucoma population^3–8^. Given the longer life expectancy of young patients, understanding the disease progression in this subgroup is crucial.

Myopia is a risk factor for the development of glaucoma^9^. However, myopic NTG is known to progress slowly. To explain this paradox, it has been hypothesized that myopic NTG exhibits relatively rapid progression in the young ages, and then become slower with cessation of eyeball growth^4,6,10,11^. However, the biphasic progression pattern has been evidenced. Thus, establishing evidence for the hypothesis would benefit many patients, and provide important insights into the natural history of myopic glaucoma.

In this study, we aimed to investigate the progression of glaucoma in myopic NTG patients of different age groups, from 20 s to focus on the very early stages of presumed myopia and glaucoma development. We also aimed to establish a quantitative record of rates by estimating the extents of axonal damage, which could be instinctively recognized as structural defects in the retinal nerve fiber layer (RNFL).

## Results

The initial cohort comprised 245 eyes of 181 patients. Excluded eyes were as follows: 10 eyes with episodes of IOP > 21 mmHg, 3 eyes with media opacities, 26 eyes with fundus tessellation, 7 eyes with diffuse RNFL loss, 2 eyes with branch retinal vein occlusion, 2 eyes with coexistent superior segmental optic hypoplasia, 3 eyes with retinal detachment surgery, 1 eye with compressive optic neuropathy, 1 eye with traumatic choroidal rupture, and 1 eye with severe epiretinal membrane that distorted the border of RNFL defect.

Finally, there were 189 eyes of 134 patients grouped as follows: 60 eyes of 44 patients in group A (initial age < 30 years), 66 eyes of 47 patients in group B (30 years ≤ initial age < 40 years), and 63 eyes of 43 patients in group C (40 years ≤ initial age < 50 years).

### Baseline and follow-up clinical characteristics

Table 1 shows the baseline and follow-up characteristics of the patients. There were no differences in refractive error, axial length, tilt ratio, tilt axis, and baseline MD between the three groups. The unadjusted mean baseline IOP was slightly higher in group A compared to group B (16.3 ± 2.0 mmHg vs 15.3 ± 1.9 mmHg, *P* = 0.045). A total of 1602 red-free fundus photographs from the total average follow-up period of 7.2 ± 3.5 years were used for analysis. The follow-up period was significantly smaller in group A (6.6 ± 4.2 years) than in group B (7.6 ± 3.3 years, *P* = 0.040), but not than in group C (7.2 ± 2.9 years, *P* = 0.093). Disc hemorrhage was observed more frequently in group C (28.3%) compared to group B (10.1%, *P* = 0.034), but not to group A (11.7%, *P* = 0.132).

**Table 1:** Baseline and follow-up characteristics of myopic NTG patients grouped by the age at first presentation.

m	Group A (age < 30)	Group B (30 ≤ age < 40)	Group C(40 ≤ age < 50)	P*	Post-hoc analysis		
					A vs B	A vs C	B vs C
Number of eyes (patients)	60 (44)	66 (47)	63 (43)	NA	NA	NA	NA
Baseline characteristics							
Sex (male, %)	21 (47.7)	24 (51.1)	21 (48.8)	0.949			
Age at first examination (years)	25.6 ± 3.3	35.8 ± 3.1	44.5 ± 3.1	** < 0.001**	** < 0.001**	** < 0.001**	** < 0.001**
Family history of glaucoma (yes, %)	6 (13.6)	2 (4.3)	5 (11.6)	0.273			
History of refractive corneal surgery (yes, %)	6 (13.6)	13 (27.7)	13 (30.2)	0.145			
Spherical equivalent (diopters)	− 6.2 ± 2.8	− 5.8 ± 2.4	− 6.0 ± 1.8	0.803			
Axial length (mm)	26.7 ± 1.0	26.4 ± 1.0	26.4 ± 0.7	0.267			
Central corneal thickness (um)	536.6 ± 42.3	522.3 ± 39.0	532.2 ± 36.3	0.227			
Tilt ratio	1.37 ± 0.24	1.34 ± 0.20	1.30 ± 0.16	0.199			
Tilt axis (°)	7.6 ± 15.0	4.2 ± 14.4	6.1 ± 13.9	0.521			
Baseline MD (dB)	− 3.90 ± 3.50	− 3.64 ± 3.72	− 3.05 ± 4.12	0.547			
Baseline RNFL defect width (°)	37.4 ± 22.8	43.4 ± 25.4	47.4 ± 33.0	0.166			
Follow-up characteristics							
Total number of examinations (times)	6.5 ± 3.2	7.6 ± 2.6	7.4 ± 2.7	**0.026**	**0.027**	0.094	0.892
Follow-up period (years)	6.6 ± 4.2	7.6 ± 3.3	7.2 ± 2.9	**0.034**	**0.040**	0.093	0.951
Untreated baseline IOP (mmHg)	16.3 ± 2.0	15.3 ± 1.9	15.2 ± 2.3	**0.024**	**0.045**	0.060	1.000
Peak IOP (mmHg)^†^	17.6 ± 2.1	17.0 ± 2.1	16.4 ± 2.3	0.050			
Mean IOP (mmHg)^†^	14.8 ± 1.7	14.6 ± 1.7	14.2 ± 2.0	0.284			
Presence of DH (yes, %)	7 (11.7)	7 (10.1)	17 (28.3)	**0.020**	1.000	0.132	**0.034**
Final MD (dB)	– 5.86 ± 5.27	− 4.74 ± 4.61	− 4.42 ± 5.18	0.155			
Final RNFL defect width (°)	57.6 ± 29.7	57.3 ± 30.2	61.1 ± 35.2	0.807			
Final number of IOP-lowering eyedrops	1.5 ± 0.9	1.3 ± 1.1	1.4 ± 0.9	0.667			

*MD* mean deviation, *DH* disc hemorrhage, *IOP* intraocular pressure, *RNFL* retinal nerve fiber layer, *NA* not applicable.

*Chi-squared test for sex and history of refractive corneal surgery, Fisher's exact test for the family history of glaucoma, and Kruskal–Wallis test for age, total number of examination, and follow-up period calculated per patient. Tukey's test with rank for post-hoc analysis. Generalized estimation equations for all other variables calculated per eye. Bonferroni correction for post-hoc analysis.

^†^IOP measurements include both treated and untreated recordings; values from observation period without treatment are included.

Significant values are in bold.

The mean IOPs were similar between groups. The final numbers of glaucoma medications were not different between the groups.

### Rates of structural glaucoma progression

The inter-observer agreement for measuring RNFL defect width was excellent (ICC 0.996, 95% confidence interval CI 0.994–0.997, *P* < 0.001). Table 2 presents the rates of RNFL defect progression in groups A, B, and C, respectively. Group A exhibited a significantly faster overall rate (3.01 ± 1.74°/year) compared to groups B and C (2.05 ± 1.55°/year and 2.06 ± 1.29°/year, *P* = 0.004 and 0.002, respectively), while no difference was observed between groups B and C (*P* = 1.000).

**Table 2:** Rates of RNFL defect progression between age-based groups and between age spans.

	Rates of RNFL defect progression (°/yr)			P*	Post-hoc analysis		
					A-B	A-C	B-C
Between age groups	Group A	Group B	Group C				
The rate calculated for the whole period	3.01 ± 1.74	2.05 ± 1.55	2.06 ± 1.29	**0.001**	**0.004**	**0.002**	1.000
The rate calculated for the initial 10 years of follow-up	3.95 ± 2.70	2.39 ± 1.64	1.98 ± 1.31	** < 0.001**	** < 0.001**	** < 0.001**	0.317
The rate calculated within the age range of 40 to 50	2.11 ± 0.84	1.76 ± 1.17	1.98 ± 1.31	0.393	0.666	1.000	0.935
Between age spans	Age period for rate calculation based on observations within the designated timeframe				[Age < 30] vs [30 ≤ age < 40]	[Age < 30] vs [40 ≤ age < 50]	[30 ≤ age < 40] vs [40 ≤ age < 50]
	[Age < 30]	[30 ≤ age < 40]	[40 ≤ age < 50]				
In group A	3.95 ± 2.70	2.40 ± 1.76	2.11 ± 0.84	** < 0.001**	**0.002**	** < 0.001**	0.856

*RNFL* retinal nerve fiber layer.

*Generalized estimation equations and Bonferroni correction for post-hoc analysis.

Significant values are in bold.

**Figure 1:**
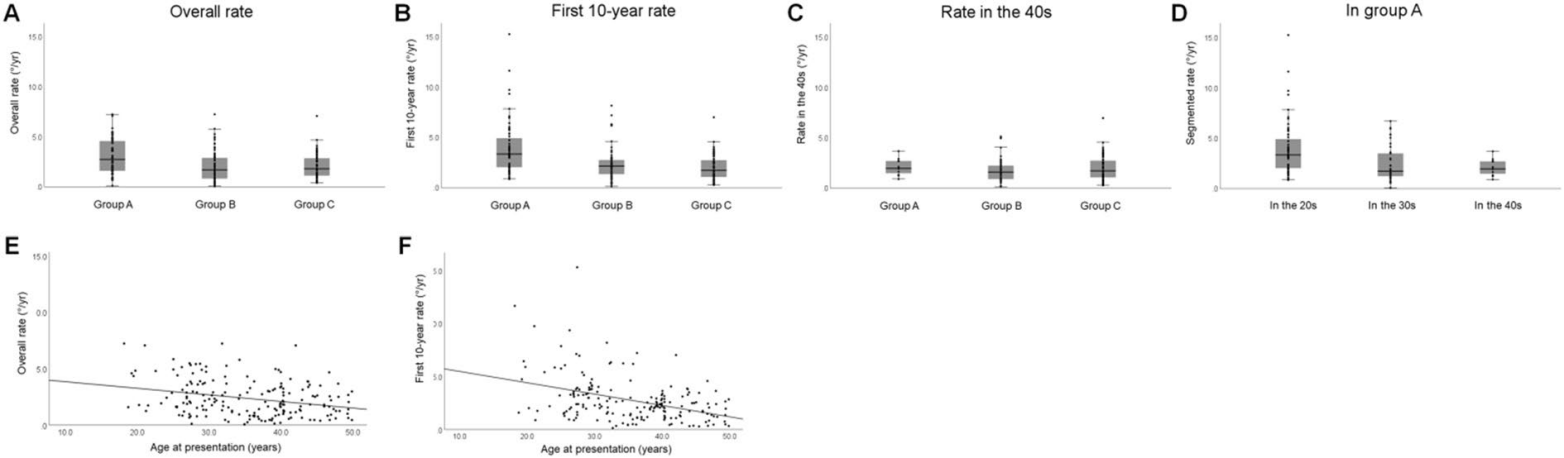
Rates of RNFL defect progression in different age groups and spans. (**A**) The overall progression rates of RNFL defect width. (**B**) The rates calculated for the first 10 years. (**C**) The rates calculated for the fourth decade. (**D**) The rates calculated by decades within group (**A**). (**E**) The relationship between age at presentation and the overall rate. (**F**) The relationship between age at presentation and the rate for the first 10 years.

The rate for the first 10 years within each group, as expected, showed more pronounced differences (3.95 ± 2.70°/year, 2.39 ± 1.64°/year, and 1.98 ± 1.31°/year, respectively) compared to the overall rate. The rates in the fourth decade did not differ significantly among the three groups, supporting the age-related difference in rates. In addition, the longitudinal comparison between age spans in group A revealed a strikingly similar result to the rates in the first 10 years; the rates for ageperiods < 30 years, 30 ≤ age < 40 years, and 40 ≤ age < 50 years were 3.95 ± 2.70°/year, 2.40 ± 1.76°/year, and 2.11 ± 0.84°/year, respectively. These results consistently indicated a faster progression in the age periods < 30 years compared to later periods. Figure 1 shows the progression rates for the three groups and age spans in group A.

There was a significant association between the age at presentation and the overall rate (odds ratio 0.943, 95% CI 0.917 – 0.970, *P* < 0.001) and the rate for the first 10 years (odds ratio 0.899, 95% CI 0.864–0.935, *P* < 0.001, Fig. 1).

**Figure 2:**
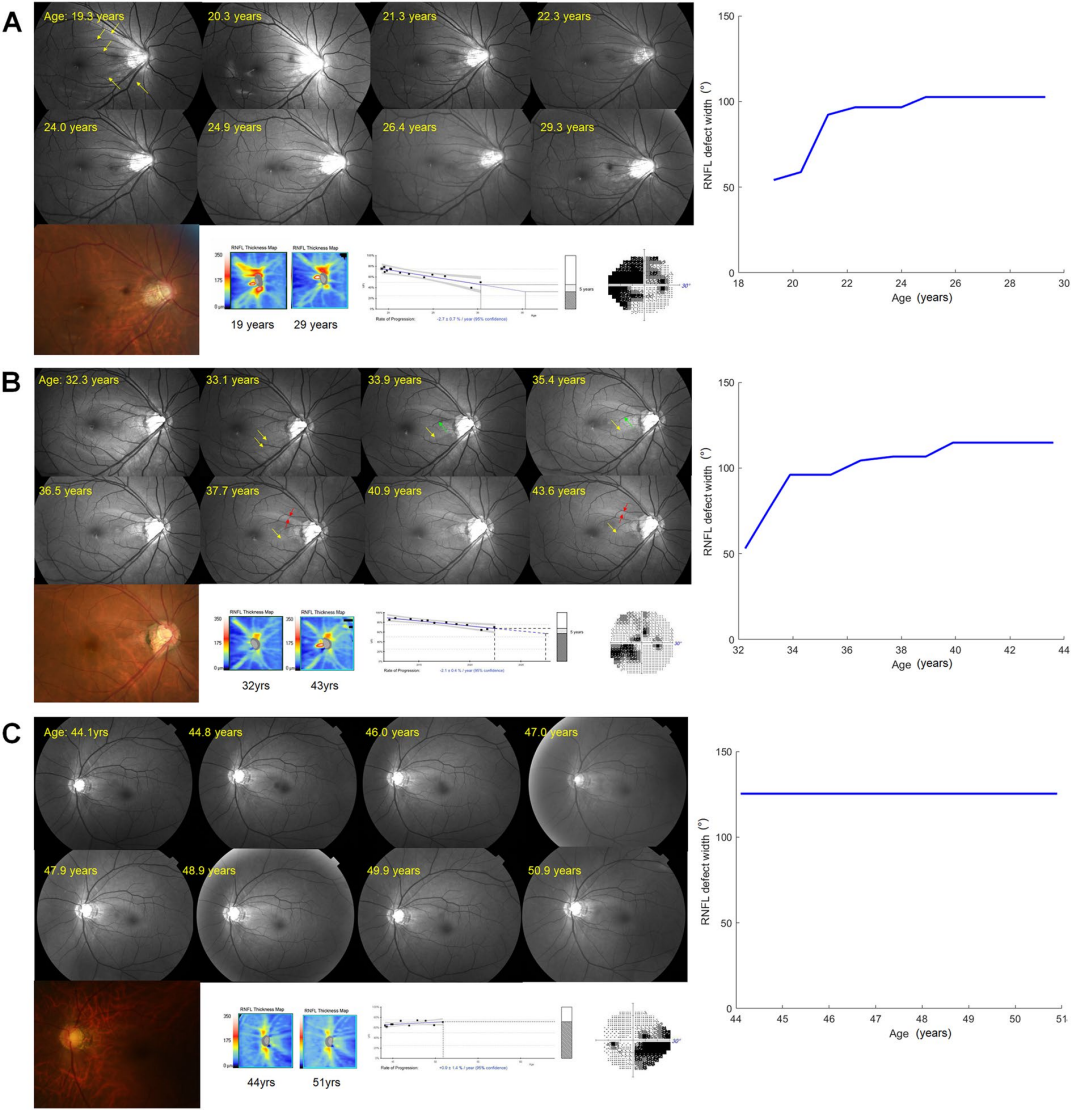
Representative cases with different age at first presentation in myopic NTG patients. (**A**) A 19.3-year-old patient with refractive error of – 9.75 D and axial length of 28.01 mm. Expansion of multiple RNFL defects (yellow arrows) accompanies progressive narrowing of areas with bright reflections that represent remaining viable axons. Early rapid progression stabilized afterwards. (**B**) A patient aged 32.3 years at first presentation, with refractive error of − 7.38 D and axial length of 26.04 mm. Progressive widening of inferotemporal RNFL defect (yellow arrows) and papillomacular bundle defect (green arrows) is observed, along with a decrease in areas with viable axons (red arrows) between the two RNFL defects. The rate of RNFL defect progression slows over time. (**C**) A patient aged 44.1 years at first presentation, who had prior implantable collamer lens (ICL) implantation, with axial length of 28.18 mm. Within the follow-up period, the RNFL defect shows no further progression from the initial examination. The images are presented in the following order: serial red-free fundus photographs (top left), final color fundus photograph (bottom left), first and last OCT thickness maps, trend-analysis for the VF index and final VF test (bottom middle), and the progression profile of RNFL defect width (right).

Figure 2 shows representative cases in each group.

### Factors associated with the rate of progression

Multivariate analysis identified age-based group and baseline MD as significant factors associated with the overall rate of RNFL defect progression (Table S1). In contrast, baseline, mean, and peak IOPs did not demonstrate a significant association.

## Discussion

In this longitudinal cohort of myopic NTG patients with different age spans, we demonstrated that in the twenties, glaucoma progresses rapidly and stabilizes afterwards. This result provides evidence for the hypothesis, for the first time, that glaucoma in myopia develops at a young age and may stabilize to some extent in older age. Moreover, the difference between age groups became more marked when calculated for the first 10 years’ period, and when calculated within the age spans of group A, highlighting the rapid progression in the twenties in cross-sectional and longitudinal aspects.

The approximate rate of wedge-shaped RNFL defect expansion was 4°/year in the 20 s, then significantly reduced by half to 2°/year. The results indicate that up to 40°-wide RNFL defect may occur in 10 years, which gives us a high alert. In these circumstances, as observed in the cases in our study, vision loss would be significant despite further stabilization of glaucoma. This contrasts with the common belief that the progression of myopic NTG is generally slow and an aggressive prognosis is not expected.

To date, researches have consistently indicated early rapid progression in myopic NTG, with an emphasis on eventual stabilization rather than initial aggressiveness^4,6,12^. Doshi et al. were first to present cases with nonprogressive glaucomatous cupping and VF defects for up to 7 years in young Chinese males associated with myopia and tilted discs (aged 25–66 years, mean 38.9 years)^4^. They suggested that the visual loss originates from progressive thinning and tilting of ONH in the young period of myopia progression that abates afterwards. Several investigators have embraced a similar concept^3–7,12^. For example, Han et al. suggested a "progression-to-stationary" pattern of VF loss in young myopic NTG patients^6,12^. The patients in the group with progression were younger compared to the group without (mean 47.8 vs 55.4 years) in 97 treatment-naïve myopic NTG patients^6^. Although the average ages in their study were much older than ours, the progression group (from 25 to 67 years) included patients in their 20 s, while the non-progression group did not (from 31 to 80 years).

Our patient group consists of exceptionally young individuals, notably younger than typically anticipated for patients with myopic NTG. The study design aimed to assess whether the onset of myopia during adolescence could be mechanistically linked to the development of glaucoma. We found that the young adulthood period was associated with a significantly faster progression rate in myopic NTG patients compared to typical old-aged periods. This result suggests a potential relationship between myopia and glaucoma within the young adulthood period, where the developmental process of myopia may directly contribute to the mechanism of glaucoma development during the same period.

Our results provide the first longitudinal evidence of the biphasic progression of NTG diagnosed in patients with myopia, but at the same time highlights that the overall visual prognosis in the young myopic NTG patients may not always be favorable.

These results may seem contradictory to the general myopic NTG patients with slow progression. We consider that there may exist two different populations regarding the impact of myopia on the development and progression of glaucoma. The patients with myopic NTG whose development occur in the late adulthood ages may be less influenced by myopia but more by age-related factors. In particular, group C with their first presentation of glaucoma over 40 years of age, are likely to have developed it in the adulthood. It is because the baseline RNFL defect width was not consistently greater in groups B and C compared to group A. Considering the fast progression of glaucoma in the young ages, at least for eyes in group C with mild RNFL defects, it is unlikely that a previous fast progression had occurred. These older patients had slow progression rates, as in the general NTG patients. In addition, we observed a higher frequency of disc hemorrhage in group C, compared to groups A and B. Additionally, the range of progression rates in the fourth decade was broader in group C than in groups A and B, and we interpreted this result as indicative of a mixed group of patients with myopia-related and typical age-related NTG.

In contrast, the pathogenic relationship between myopia and glaucoma may be particularly strong in young ages. The possibility of myopic changes contributing to glaucomatous damage is supported by the dose–response relationship between the degree of myopia and glaucoma^13^, as well as the coincidence in the period of myopia development and, hypothetically, the development of myopic glaucoma. Myopia develops and progresses during adolescence and teenage years^10,11^. Glaucoma develops at younger ages than the general glaucoma population in myopic patients, such as in the 20 – 30 years^3–7^, 18–28 years^8^ and even teenage years^14^, Therefore, in young myopic individuals who are developing glaucoma, myopic changes, such as the deformation of ONH structures, may lead to accelerated axonal loss in the context of glaucomatous damage.

In addition, we noted the temporal gap of at least a decade between the time of presumed myopia stabilization, in teenagers^10,11^ and glaucoma stabilization, in the 30 s. The reasons why glaucoma progression continues for several years after the completion of axial growth are unknown. We speculated that the gradual remodeling of ocular tissues may lead to an altered biomechanical microenvironment that may persist for some time. With aging, the effect may diminish as age-related changes in material properties, such as the stiffness of the sclera and ONH, may override those of myopization. Further investigation is warranted to understand the significance, including the longitudinal changes in the biomechanical features, such as ONH strain^15^, in eyes with myopia and glaucoma.

Despite the obvious and consistent results, one may concern that measurements from red-free fundus photographs may be less appropriate than those from OCTs with objective measurements of 3D RNFL thickness. Specifically, our method allows for the evaluation of only the width, not the depth. Nevertheless, we would like to address several clinical merits in the measurements in red-free fundus photographs. First, it is a morphologic indicator that can be intuitively recognized. The changes presented as the wedge-shaped RNFL defect provide more instinct message than numerical values for the global RNFL thickness in OCTs. Besides, it allows us topographic localization of the damage, which is critical for the quality of the patient’s VF. Second, OCT results may be affected by signal strength, the placement of the scan center, and inter-examination variabilities, which are relatively absent in photographs. Third, red-free fundus photographs in highly-pigmented Asian eyes clearly show the borders of localized RNFL defects, such as in our study^16^. Many studies in Asian eyes have therefore employed the measurement of RNFL defect width to assess glaucoma progression^17–21^. In addition, due to the resolution, OCT may be less sensitive than red-free fundus photographs in detecting subtle changes, especially in myopic eyes^19,22–28^. Lastly, flicker analysis, which at least guarantees detection of a significant change, was performed to overcome the subjectivity in measurements. Therefore, direct morphological measurement of RNFL defects may benefit from intuitive messaging and anatomical relevance^23^ in study population such as ours, providing clinically useful estimates, at least in demonstrating rapid progression in young adulthood.

Nevertheless, due to the limitations of red-free photographs, assessing the RNFL defect solely through red-free fundus photographs without the aid of OCT may not be practical. In clinical settings, OCT is widely adopted as an objective and highly useful tool for patient monitoring. Therefore, it would be more appropriate to consider and utilize the insights from our study, particularly the rates of RNFL defect width progression, in conjunction with the results provided by OCT.

In our study, IOP was not significantly associated with glaucoma progression rate during the follow-up period. Despite the well-known significance of IOP in glaucoma progression, even in NTG patients, we speculate that our study population may differ from typical glaucoma patients, where the mechanism of glaucoma may be distinctly associated with biomechanical conditions related to myopic changes, rather than IOP. Consequently, the influence of IOP may be less pronounced than in the general glaucoma population. Additionally, as all IOP measurements were under 21 mmHg, the limited range of IOP may also have contributed to the insignificant relationship between IOP and progression rate.

There are several limitations of this study. Firstly, it was a retrospective study with a relatively small sample size. However, obtaining long-term longitudinal data for young patients is a challenge, making our study valuable despite the limited sample size. Secondly, we included a subset of patients who had undergone refractive corneal surgery. However, the impact of such surgery on glaucoma progression remains unclear, and it was not a significant risk factor for glaucoma progression in one study^29^. Thirdly, treatment could have affected the results. However, aggressive treatment was administered to all patients in the young age groups due to their long life expectancy, while some adult patients were recommended a medication-free observation period. Therefore, the faster progression in the younger group could have been even more pronounced, and treatment did not seem to introduce bias into the results. The IOP was also maintained at a similar level across all three groups during the follow-up period. Although a slightly lower peak IOP was observed in group C compared to groups A and B, this difference (approximately 1 mmHg) was not sufficient to explain the disparity in progression rates. Further investigations are warranted to evaluate the effect of IOP control in these patients. Also, the shorter, not longer, follow-up period in group A may not interfere with the faster rate than in groups B or C. Finally, all IOP recordings were based in outpatient clinics. Therefore, there is a possibility of undetected IOP elevation, such as in nocturnal IOP spikes. A comprehensive 24-h evaluation of IOP would be beneficial to fully comprehend the IOP profile of the patients and facilitate a more precise diagnosis between NTG and high-tension glaucoma.

In conclusion, our study provides the first longitudinal evidence of rapid myopic NTG progression in young adulthood and the stabilization in older age. Clinicians should pay attention to the potentially aggressive course of glaucoma, especially in younger patients, despite the generally slow progression of myopic NTG in adulthood.

## Methods

This was a retrospective longitudinal cohort study. Data of patients who visited Samsung Medical Center (Seoul, South Korea) between February 2007 and January 2023 were used. This study followed all guidelines for experimental investigation in humans, was approved by the Samsung Medical Center Institutional Review Board (#2023-04-074) and adhered to the tenets of the Declaration of Helsinki. The IRB waived the requirement for informed consent due to the retrospective study design and the absence of potential harm to study subjects.

### Eligibility criteria

Patients with myopic NTG who had at least three glaucoma examinations were included. Patients were categorized into three groups based on their age at the first presentation: group A (< 30 years), group B (≥ 30 and < 40 years), and group C (≥ 40 and < 50 years). Myopia was defined as a spherical equivalent of less than − 0.5 diopters (D)^2^. Because young NTG patients diagnosed under 40 years of age are relatively rare, all possible candidates for the eligibility criteria were enrolled.

The diagnosis of NTG was based on the characteristic optic disc changes, RNFL defects, and corresponding VF defects. Specifically, wedge-shaped sectoral RNFL defects in the superotemporal area, inferotemporal area, or in the papillomacular bundle, neuroretinal rim thinning or notching in the corresponding location to the RNFL defects, absence of optic disc pallor, and topographically corresponding glaucomatous VF defects were required. We did not include eyes with VF changes only, without characteristic neuroretinal rim changes and RNFL defects. Additionally, the patient needed to have an open anterior chamber angle and untreated intraocular pressure (IOP) ≤ 21 mmHg. IOP assessment was based on records performed in the outpatient clinic.

Exclusion criteria comprised patients with (1) IOP > 21 mmHg during the follow-up period, (2) media opacities such as corneal opacity, cataract, or vitreous opacity, (3) without clear recognition of RNFL defect boundaries, from factors such as fundus tessellation or diffuse involvement of RNFL, and (4) concurrent ocular or systemic diseases that could impact RNFL and VF tests.

### Comprehensive ophthalmic evaluation

The initial examination included measurement of visual acuity and refraction, Goldmann applanation tonometry, slit-lamp biomicroscopy, and gonioscopic examination. Dilated stereoscopic examination of the optic nerve head (ONH), color and red-free fundus photography (Topcon, Paramus, NJ), central 30-2 static automated perimetry using the Humphrey Field Analyzer (HFA), ultrasound pachymetry (Tomey SP-3000, Tomey Ltd., Nagoya, Japan), and spectral-domain optical coherence tomography (OCT) with Cirrus HD-OCT (Carl Zeiss Meditec, Dublin, CA) were performed annually or as determined by the physician.

### Evaluation of the structural glaucoma progression rate

We estimated the rate of structural glaucoma progression by analyzing the rate of change in the extent of RNFL defect. We measured the width of RNFL defect in red-free fundus photographs. We used red-free fundus photographs because the fundi of the Asian eyes are highly pigmented, which enable a clear recognition of the border of RNFL defects^16^. Nevertheless, to overcome the limitation in the subjective measurement of RNFL defect width, we first created an overlay image by superimposing a complete set of red-free fundus photographs by vascular landmarks. Second, meticulous flicker analysis was performed before each measurement for better accuracy and sensitivity of comparison. Nevertheless, this methodology has limitations as it only allows for the evaluation of the width, not depth, of an RNFL defect. However, a portion of the patient data with OCT was unavailable due to the recent adoption of OCT in our clinic; we used traditional examinations for a more extended period of analysis.

**Figure 3:**
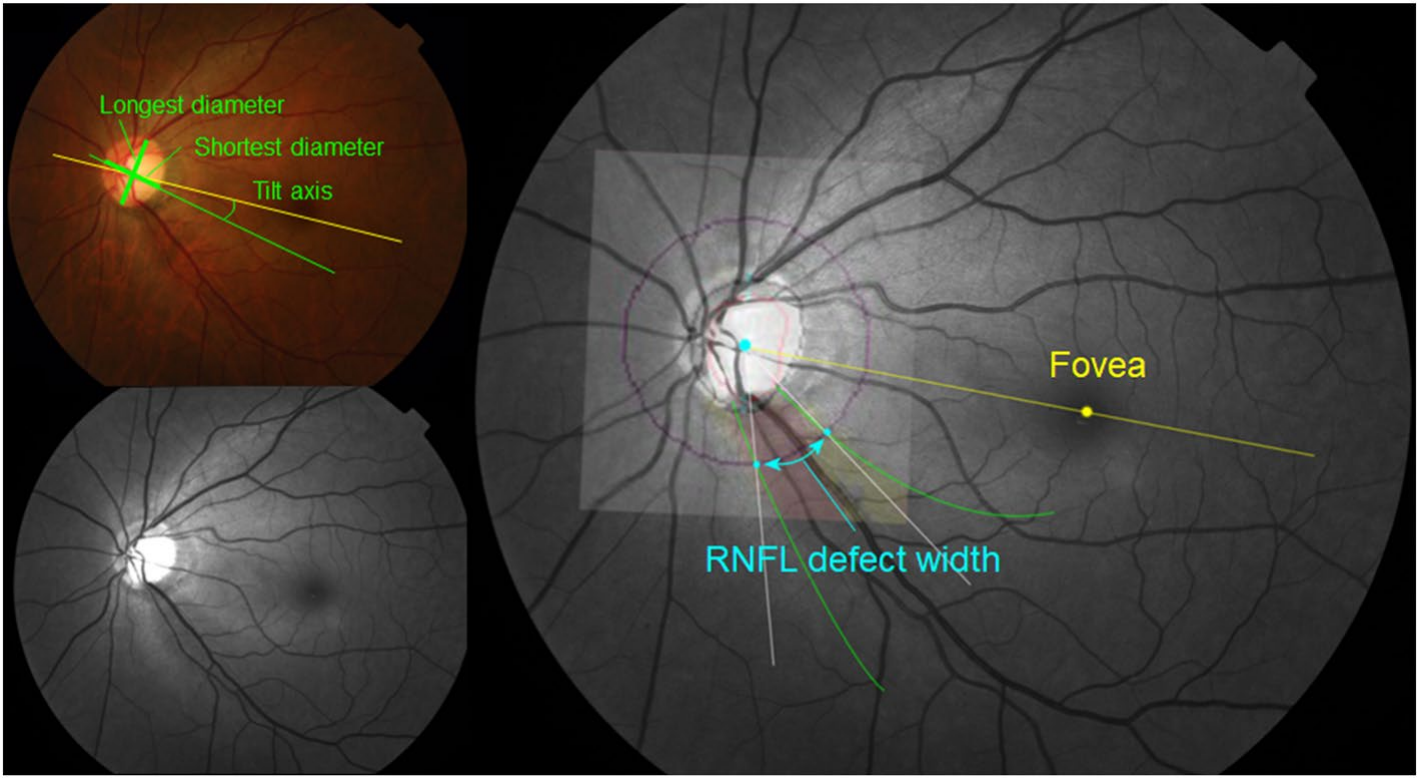
Measurement of RNFL defect width. Measurement of the width of the retinal nerve fiber layer (RNFL) defect by the intersections between the RNFL defect border and the 3.5 mm diameter RNFL thickness scan circle of Cirrus optical coherence tomography (OCT) superimposed on the red-free fundus photograph using vascular landmarks.

We measured the angular circumferential width of the RNFL defect by determining the intersections between the RNFL defect border and the 3.5 mm diameter RNFL thickness scan circle of Cirrus OCT, which was superimposed identically (Fig. 3). The total sum of all measurements was used for cases with multiple RNFL defects. A software Adobe Photoshop (Adobe, San Jose, CA, USA) was used.

### Measurement of tilt ratio and tilt axis

We measured the tilt ratio, defined as the ratio between the longest diameter and shortest diameter of the optic disc^3^. Tilt axis was defined as the deviation of the shortest diameter of the disc from the line connecting the fovea and the center of the disc (Fig. 3)^3^. Positive angles indicated the inferior direction. All measurements were performed using ImageJ software (National Institutes of Health, Bethesda, MD).

### Patient management

Considering the young age and long life expectancy, all patients in groups A and B were treated with IOP-lowering eyedrops at diagnosis. For patients in group C, we observed the patients without treatment when the VF defect was mild and confined to peripheral areas; immediate treatment with eyedrops was initiated upon detecting glaucoma progression during follow-up. The treatment goal aimed for a 20–30% reduction in IOP. In cases with a stable glaucoma course, additional eyedrops were not prescribed even if the target reduction was not fully achieved. Treatment intensification was considered when consistent elevations of IOP in the high teens were observed or when signs of glaucoma progression were detected. For patients with fluctuating or moderately elevated IOPs, we adjusted treatment and follow-up schedules based on the severity of glaucoma, corneal thickness, and adherence to eyedrops. Glaucoma progression was defined by corresponding changes in neuroretinal rim, RNFL defect in red-free fundus photographs, OCT thickness measurements, and VF tests. VF progression adhered to modified Anderson’s criteria^30^, requiring a significant deterioration from the baseline pattern deviation with three adjacent points depressed by ≥ 5 dB, with at least one point depressed by 10 dB in consecutive VF tests. Nevertheless, treatment was also intensified in eyes without VF progression when the structural progression was obvious. Selective laser trabeculoplasty or surgeries were not performed on study patients, as all IOP recordings remained below 21 mmHg.

### Statistical analysis

We assessed inter-observer reproducibility of RNFL defect width measurements using intraclass correlation coefficients (ICCs) with 95% confidence intervals (CIs) based on 106 measurements from 11 eyes. For inter-group comparisons, we used the chi-squared test for sex and history of refractive corneal surgery, Fisher’s exact test for family history of glaucoma, and Kruskal–Wallis test for age, total number of examinations, and follow-up periods per patient. Post-hoc comparisons were conducted using Tukey’s test with rank. We used generalized estimation equations (GEEs) for all other variables calculated per eye to account for the correlation of eyes within patients. For eyes that underwent refractive surgery, the refractive values were treated as missing data. We estimated the rate of RNFL defect width progression for each eye using linear mixed-effects modeling with RNFL defect width as the response, time as a fixed effect, and random intercept and slope for each eye nested within each patient. We estimated segmented rates of RNFL defect width progression using piecewise linear mixed-effects modeling, divided into three pieces representing the age periods < 30 years, 30 ≤ age < 40 years, and 40 ≤ age < 50 years. Only measurements performed during each age period in different patients were utilized to estimate rates confined within each age period. We performed univariate and multivariate analyses to identify parameters associated with the rate of RNFL defect width progression. All statistical analyses were performed using SAS version 9.4 (SAS Institute, Cary, NC), and *P* < 0.05 were considered statistically significant.

### Supplementary Information

Supplementary Table S1.

## Data Availability

The datasets used and/or analyzed during the current study available from the corresponding author on reasonable request.
