# Supplementary material for: Different glaucoma progression rates by age groups in young myopic glaucoma patients
Source: Sci Rep. 2024 Jan 31;14:2589. doi: 10.1038/s41598-024-53133-w (PMC10830449; doi:10.1038/s41598-024-53133-w)
Supplement: Supplementary file 1 — Supplementary Table S1. [file 41598_2024_53133_MOESM1_ESM.docx]

**Supplementary Material**

**Table S1. Factors associated with the progression rate of RNFL defect**

|  | Univariate analysis | | | Multivariate analysis model 1 | | | Multivariate analysis model 2 | | | Multivariate analysis model 2 | | |
| --- | --- | --- | --- | --- | --- | --- | --- | --- | --- | --- | --- | --- |
| Parameter | β | 95% CI | *P* | β | 95% CI | *P* | β | 95% CI | *P* | β | 95% CI | *P* |
| Age group |  |  | **0.001** |  |  | **0.014** |  |  | **0.004** |  |  | **0.004** |
| A vs B | 0.958 | 0.372 – 1.544 | **0.004** | 0.985 | 0.181 – 1.789 | **0.010** | 0.933 | 0.200 – 1.666 | **0.007** | 0.953 | 0.204 – 1.702 | **0.007** |
| A vs C | 0.952 | 0.406 – 1.499 | **0.002** | 0.712 | -0.052 – 1.477 | 0.078 | 0.889 | 0.189 – 1.589 | **0.007** | 0.918 | 0.203 – 1.634 | **0.007** |
| B vs C | -0.005 | -0.482 – 0.471 | 1.000 | -0.273 | -0.859 – 0.313 | 0.799 | -0.044 | -0.639 – 0.552 | 1.000 | -0.035 | -0.618 – 0.549 | 1.000 |
| Sex | 0.128 | -0.328 – 0.583 | 0.582 |  |  |  |  |  |  |  |  |  |
| Total number of examinations | -0.074 | -0.142 – -0.006 | **0.033** |  |  |  |  |  |  |  |  |  |
| Follow-up period | -0.064 | -0.126 – -0.001 | **0.046** |  |  |  |  |  |  |  |  |  |
| Family history of glaucoma | 0.207 | -0.608 – 1.022 | 0.619 |  |  |  |  |  |  |  |  |  |
| Previous refractive surgery | 0.244 | -0.264 – 0.753 | 0.346 |  |  |  |  |  |  |  |  |  |
| Baseline MD | -0.069 | -0.128 – -0.011 | **0.020** | -0.102 | -0.159 – -0.044 | **<0.001** | -0.065 | -0.124 – -0.006 | **0.031** | -0.067 | -0.126 – -0.007 | **0.027** |
| Disc hemorrhage | 0.050 | -0.482 – 0.582 | 0.854 |  |  |  |  |  |  |  |  |  |
| Axial length | 0.285 | 0.002 – 0.569 | **0.049** | 0.123 | -0.178 – 0.424 | 0.423 | 0.200 | -0.084 – 0.485 | 0.168 | 0.195 | -0.090 – 0.480 | 0.180 |
| Spherical equivalent | -0.103 | -0.224 – 0.018 | 0.094 |  |  |  |  |  |  |  |  |  |
| Central corneal thickness | -0.003 | -0.009 – 0.002 | 0.245 |  |  |  |  |  |  |  |  |  |
| Tilt ratio | 0.302 | -0.977 – 1.580 | 0.644 |  |  |  |  |  |  |  |  |  |
| Tilt axis | 0.005 | -0.009 – 0.020 | 0.465 |  |  |  |  |  |  |  |  |  |
| Baseline IOP | 0.049 | -0.065 – 0.163 | 0.401 | -0.015 | -0.122 – 0.091 | 0.779 |  |  |  |  |  |  |
| Mean IOP | -0.023 | -0.161 – 0.115 | 0.743 |  |  |  | -0.064 | -0.196 – 0.069 | 0.348 |  |  |  |
| Peak IOP | -0.046 | -0.150 – 0.059 | 0.389 |  |  |  |  |  |  | -0.060 | -0.162 – 0.043 | 0.256 |
| Baseline RNFL defect width | 0.003 | -0.005 – 0.011 | 0.472 |  |  |  |  |  |  |  |  |  |

RNFL=retinal nerve fiber layer; OR=odds ratio; CI=confidence interval; MD=mean deviation; IOP=intraocular pressure.

P value by generalized estimation equations.
